# Profiling and validation of individual and patterns of *Chlamydia trachomatis*-specific antibody responses in trachomatous trichiasis

**DOI:** 10.1186/s13071-017-2078-8

**Published:** 2017-03-13

**Authors:** Harry Pickering, Sarah E. Burr, Tamsyn Derrick, Pateh Makalo, Hassan Joof, Richard D. Hayward, Martin J. Holland

**Affiliations:** 1 Clinical Research Department, London School of Hygiene and Tropical Medicine, Keppel Street, London, UK; 2 Disease Control and Elimination Theme, Medical Research Council, The Gambia Unit, Fajara, Banjul, Gambia; 3 Institute of Structural and Molecular Biology, Birkbeck and University College London, Malet Street, London, UK

**Keywords:** *Chlamydia trachomatis*, Trachoma, Trachomatous scarring, Trachomatous trichiasis, Microarray, Antibodies

## Abstract

**Background:**

Ocular *Chlamydia trachomatis* (Ct) infection causes trachoma, the leading infectious cause of blindness. A Ct D/UW3 proteome microarray and sera from Gambian adults with trachomatous trichiasis (TT) or healthy matched controls previously identified several novel antigens, which suggested differential recognition in adults with TT.

**Methods:**

We re-analysed this serological microarray data using more robust microarray analysis techniques accounting for typical problems associated with highly dimensional data. We examined the Ct-specific antibody profile concerning the overall diversity of responses, antigen expression stage and cellular localisation of antigens. We tested differentially recognised antigens by further serological testing of the screened sera and used larger independent sample sets for validation.

**Results:**

Antibody responses identified High-Performance on antigens expressed early and late in the Ct developmental cycle and those secreted or localised to the outer membrane. Eight antigens were preferentially recognised by scarred individuals and one antigen by healthy individuals. Three of these antigens, two associated with scarring (CT667 and CT706) and one healthy-associated (CT442), were not associated with the presence or absence of scarring following specific serological testing of the arrayed sera and sera from larger, independent case-control cohorts.

**Conclusions:**

This study identified focussed Ct-specific antibody profiles targeting proteins expressed during entry and exit from cells and localised to interact with the host. A small panel of antibody responses could discriminate between adults with and without TT in a trachoma-endemic community. Heterogenous responses in the independent validation of these antibody targets highlighted the need for large sample sizes, clearly defined clinical phenotypes and follow-up work.

**Electronic supplementary material:**

The online version of this article (doi:10.1186/s13071-017-2078-8) contains supplementary material, which is available to authorized users.

## Background

Trachoma, caused by ocular infection with *Chlamydia trachomatis* (Ct), is the leading infectious cause of blindness worldwide. Ocular infections with Ct affect the epithelial cells of the conjunctiva [1], with repeated infection in endemic areas causing a chronic keratoconjunctivitis [2, 3]. Chronic and repeated episodes of infection and disease in children induce changes in the tissue underlying the conjunctiva, leading to deposition of scar tissue. Progression of this scarring pathology can lead to trichiasis (TT), corneal opacities (CO) and blindness [4]. The majority of people in trachoma-endemic communities do not progress to these latter stages of trachomatous disease and pathology varies considerably within those that do progress. This heterogeneity seems, in part, due to the impact of prolonged infection and inflammation; however other risk factors have also been identified including age, gender, dry eye and non-chlamydial bacteria [5–13].

Levels of IgG antibodies against Ct elementary bodies (EBs) are significantly higher in individuals with scarring trachoma [14–16]. Since frequent and persistent infections are associated with scarring, this suggests the development of these antibodies does not protect from progression. One of these studies found higher levels of antibody against the Ct antigen HSP60 in scarring individuals independent of responses against EBs, implying HSP60 is not simply a marker of increased exposure. This association of anti-HSP60 antibodies was not consistent between studies of trachomatous scarring (TS) and trachomatous trichiasis (TT) [17, 18]. However, one of these studies did demonstrate that IgG antibodies against another Ct antigen, CPAF, were significantly increased in TT [18]. It is unclear whether the scarring-associated antibody responses identified in trachoma-endemic communities are common throughout the population or if they are directly involved in the scarring process. It is possible via opsonisation that anti-Ct antibodies could facilitate greater Ct infectivity in young children promoting frequent and prolonged infections that are known to be a risk factor for TS/TT. Equally, they may be coincidental serological markers of infection.

The last decade has seen the exploitation of protein-based screens of human serum to document the complete profile of antibody responses stimulated by an infection [19]. This has streamlined the identification of diagnostic and vaccine candidates, leading to faster progression and evaluation of individual targets. For *Plasmodium falciparum* and malaria, targeted panels of proteins have been screened to identify immunity-associated antigens [20] and antigens associated with particular stages of infection [21]. Similar studies have been applied to some bacterial species including *Mycobacterium tuberculosis* [22] and 30 causative organisms of tropical infectious diseases [23].

There have been six published studies that have screened human serum against microarrays of Ct antigens to define serological responses [24–27], two of these simultaneously investigated T-cell responses [28, 29]. A comparison of these studies (summarised in Additional file 1: Table S1), identifies some commonly recognised antigens. However, the majority were identified in only one or two studies. This variation likely represents methodological differences and heterogeneity in immune responses targeting Ct antigens. A similar murine study found more focussed serological responses in C57/BL6 mice which are more resistant to urogenital chlamydial infection than BALB/c and C3H/HeN mice, suggesting variations in susceptibility to infection are partly related to antibody profiles [30].

Previously, serum from individuals with TT was used to screen a Ct D/UW3 proteome array of 908 genomic and plasmid ORFs [31]. This study used 61 cases of trichiasis (TT) and 61 age, sex and location-matched controls with normal healthy (healthy) eyes collected in The Gambia between May 2006 and February 2009. Thirty-four cases and 25 controls were screened on the serovar D Ct proteome array [24, 25]. Ten antigens were recognised by > 50% of the 59 samples tested. Four antigens were preferentially recognised by those with TT and eight by healthy controls (Table 1). However, there were some limitations in the study including in both the wet and dry lab study methodologies. The small sample size, lack of global normalisation and inadequate measures to control against false discovery each contributed to a high chance of error [32].

**Table 1:** Participant demographics from the study of TS and matched controls from The Gambia in 1995. Age (generalised linear model) and gender (Fisher’s exact test) were compared between healthy controls and scarred cases, associated *P*-values are indicated

	Healthy controls	Scarred cases	*P*-value
Number	116	115	na
Age in years (95% CI)	37.50 (7.00–65.00)	38.00 (7.00–65.75)	0.900
Female, *n* (%)	84 (72.41)	80 (69.57)	0.633

*Abbreviation*: *na* not applicable, *CI* Confidence intervals, *n* number

Here, we report the results of a more robust biostatistical analysis of data generated from this array. We then determined differences in response diversity, richness and evenness by employing standard metrics used in ecology and analysis of 16S-amplicon community surveys. We additionally utilised sera from independent studies of TS/TT to demonstrate the need for validation of antibody responses determined using large-scale screening of small numbers of participants.

## Methods

### Study participants

Sera were taken from three clinical cohorts collected in The Gambia between 1995 and 2011. In 1995, 153 people with evidence of TS by clinical examination were recruited alongside age, sex and village-matched controls with normal eyes from Kaur Health Centre and the villages of Jali and Berending (SCC729). A 1 ml sample of venous blood was taken from each person to obtain serum [33, 34]. A total of 231 serum samples were re-tested from this study in the present analysis (Table 1).

Between May 2006 and February 2009, 61 people with evidence of TT and corneal opacities (CO) by clinical examination were recruited alongside age, sex and village-matched controls with normal eyes from the Western, Central and Lower River Regions of The Gambia (SCCL2006.10). A sample of venous blood was taken from each person to obtain serum. Details of the cohort and the collection are available in Lu et al. [31]. A total of 116 of these archived sera were available for testing by the present study (Table 2). Thirty-four cases and 25 controls were screened on the serovar D Ct proteome array described previously [31]. Briefly, 59 individuals with high titres of anti-Ct ocular serovar antibodies were selected, removing individuals with presumed low anti-Ct antibody levels. Serum samples were screened against a GST-fusion protein micro-array of 908 ORFs from Ct D/UW3.

**Table 2:** Participant demographics from the study of TT and matched controls collected between May 2006 and February 2009 in The Gambia. Age (generalised linear model) and gender (Fisher’s exact test) were compared between healthy controls and scarred cases, associated *P*-values are indicated

	Healthy controls	Scarred cases	*P*-value
Number	58	58	na
Age in years (95% CI)	55.50 (30.43–73.73)	60.00 (34.00–77.88)	0.199
Female (*n* [%])	40 (68.97)	39 (67.24)	0.842

*Abbreviation*: *na* not applicable, *CI* Confidence intervals, *n* number

In 2011 (SCC1274), 90 people with evidence of TS and TT by clinical examination were recruited alongside age, sex and village-matched controls with normal eyes from multiple rural regions [35] (Table 3). A 1 ml sample of venous blood was taken from each person to obtain serum, sera from all 90 patients were available for testing in the present study.

**Table 3:** Participant demographics from the study of TT/CO and matched controls from The Gambia in 2011. Age (generalised linear model) and gender (Fisher’s exact test) were compared between healthy controls and scarred cases, associated *P*-values are indicated

	Healthy controls	Scarred cases	*P*-value
Number	38	52	na
Age in years (95% CI)	19.50 (1.00–39.00)	20.50 (3.55–37.73)	0.224
Female, *n* (%)	30 (78.95)	45 (86.54)	0.343

*Abbreviation*: *na* not applicable, *CI* confidence intervals, *n* number

### Normalisation, filtering and positivity

The raw optical density data from the microarray (Additional file 2: Table S2) was transformed by inverse hyperbolic sine transformation and normalised by mean-centring, these techniques were determined as the most suitable ‘normalisation’ step using relevant rank deviation (RRD) [36]. Post-normalisation, the global median of the data, was calculated, individual antigens whose median was lower than the global median were excluded.

Lu et al. defined a positive response as an optical density (OD) equal to or greater than two standard deviations above the mean from the relevant 96-well plate [31]. However, there is no consensus method to differentiate at which point a response or value is judged to be significantly positive in serological microarrays. In this reanalysis, we, therefore, tested several different methods to identify positive-negative breakpoints in the distribution of the data [37]. These were: k-means clustering, k-medoids clustering, fuzzy c-means clustering, hierarchical clustering and mixture modelling. These were tested allowing for 2 to 10 clusters. Maximisation of between cluster variance was favoured over minimisation of within-cluster variance. Silhouette analysis was selected to quantify the effectiveness of clustering methods. The average silhouette width of each antigen was used to determine the appropriateness of the cluster configuration [38]. The mean of each silhouette per antigen gave the average silhouette width, which illustrates how tightly the data were plate array. To determine positive responses, two Lyophilized were identified and the method that resulted in the highest average silhouette width for each antigen identified. Data points clustered with the maximum OD/signal intensity point of each antigen were considered positive and the opposing cluster negative.

### Diversity metrics

Ecological measures of diversity rely on species breadth/richness, the total number of species in a sample, and species diversity, which additionally incorporates the relative abundance of each species. We treated antigens as species, abundance as the response to each antigen and the samples were either the entire data set or the data set split into dichotomous outcome variables. These definitions are based on the assumption that responses on the array correlate with abundance of antibodies in each sample. A normalised OD = 1 some as one arbitrary unit of an antibody. This means if a response to an antigen is twice as high in one sample compared with another, antibodies are twice as abundant in that individual.

Breadth was defined as the number of antigens to which each had a positive response. For the remaining measures examining diversity, existing methods were exploited by utilising the continuous OD/signal intensity values. This was deemed more appropriate as an assumption of these methods was that individuals within a species are equivalent [39, 40], in this analysis the species are antigens, and positive responses to them are not equal.

We utilised two different measures of diversity to improve the reliability of the results. Shannon’s entropy (*H*) and Simpson’s index (*D*) [40]. Higher values for both indicate increased diversity and greater evenness. High values of *H* mean that an unknown individual could belong to any species. In our context, this means one unit of antibody in a sample could be targeted against any antigen because responses in the sample are equivalent [41]. High values of *D* mean that two randomly chosen individuals are likely to be from different species. In our context, this means two separate units of antibody from the array are unlikely to be targeted against the same antigen due to evenness of the responses [41].

### Recombinant protein expression and peptide synthesis

Pgp3, CT667 and CT706 GST-fusion Ct constructs were provided by Professor Guangming Zhong (UT Health Science Center, San Antonio, TX) and were produced as previously described [24]. Briefly, 1 L of 2x YT media was inoculated with frozen stocks of transformed *E. coli* and incubated at 37 °C overnight at 230 RPM. Overnight cultures were diluted into 2× YT media and incubated at 37 °C at 230 RPM until OD 600 reached 0.6 to 1.2. IPTG was added at a concentration of 200 μM to induce expression and incubated at 30 °C for three h. Cultures were centrifuged at 4 °C for 30 min at 4000× *g*, and the harvested cells were resuspended in 1:20 culture volume of phosphate-buffered saline (PBS) with protease inhibitors. The bacteria were lysed by mechanical cell disruption followed by centrifugation at 4 °C for 30 min at 4000× *g* to remove aggregated material and cell debris. Relevant detergents were added, 1% (v/v) Triton X-100 for Pgp3 and CT667 and 1% (w/v) ASB-14 for CT706, and the samples incubated at room temperature for 2 h on a roller. The samples were centrifuged at 4 °C for 1 g at 12,000× *g*, and the supernatants containing soluble proteins and stored at -20 °C.

To affinity purify proteins 1:2000 culture volume of a 50% slurry of glutathione Sepharose beads was added to the soluble protein samples to bind the GST moiety and the mixture incubated at room temperature for 2 h on a roller. The samples were passed down a 5 ml polypropylene column leaving a bed of GST-fusion bound beads. To cleave the GST-fusion before purification, the beads were washed. The correlation times with 5 ml cleavage buffer before adding 1:4000 culture volume cleavage buffer with 8% (v/v) PreScission Protease (GE Healthcare Life Sciences, Little Chalfont, UK) and incubated at 4 °C overnight. The eluates were collected, and the elution step repeated twice. Cleaved recombinant proteins were further purified by size-exclusion based gel-filtration chromatography using a 35 ml Superdex 200 column (GE Healthcare Life Sciences) with a ÄKTApurifier.

Biotinylated peptides of CT442 were produced by thinkpeptides (ProImmune, Oxford, UK), using sequences from Ct D/UW3. Purity High-Performance by High Performance Liquid Chromatography (HPLC), the minimum accepted was 80%. Lyophilized peptides were resuspended in distilled H_2_O, aliquoted and stored at -80 °C. The CT442 peptide sequence (amino acid 135–150) had been previously identified as immunogenic (Dr Bernhard Kaltenboeck, personal communication) [42].

### ELISA testing

The initial ELISA protocol was adapted from methods used previously in trachoma or urogenital Ct infection [18, 24]. The protocol was validated by comparing the results with two published Pgp3-based ELISA protocols [43, 44]. Recombinant proteins were diluted to 1 μg/ml in coating buffer (0.05 M carbonate-bicarbonate in PBS at pH 9.6) and 50 μl/well added to the first 90 wells of Immulon 4 HBX microtitre plates (Fisher Scientific, Loughborough, UK). The positive control antigen Pgp3 was similarly diluted and added to the remaining 6 wells of each plate as a positive control. Antigens were bound at 4 °C overnight. Sera/plasma were diluted 1:500 in blocking buffer [0.05% (v/v) Triton X-100 and 2.5% (w/v) skimmed milk in PBS at pH 7.5]. Four hyper-immune control sera were pooled and diluted 1:100 and then serially diluted 1/5, 5 times. After 30 min agitation, diluted samples were stored at 4 °C overnight.

The following day plates were inverted and washed twice with washing buffer [0.05% (v/v) Triton X-100 in PBS at pH 7.5] and blocked at room temperature for 4 h. After 2 washes 100 μl test sera in triplicate and control sera were added and incubated at room temperature for 4 h. After 4 washes, 100 μl anti-human IgG-peroxidase antibody diluted 1:30000 in blocking buffer was added per well and incubated at room temperature for 1 h. After a final 4 washes, 100 μl 1-Step Ultra TMB-ELISA substrate (Fisher Scientific, Loughborough, UK) were added per well and incubated at room temperature for 10 min. The reaction was stopped by addition of 100 μl 2 M sulphuric acid per well, and the plate read at OD 450 nm for detection and 700 nm for background correction.

Biotinylated peptides were tested using a modified protocol. Stocks of streptavidin were diluted to 5 μg/ml in H_2_O, and 100 μl/well added to the first 90 wells and Pgp3 was added to the remaining 6 wells on each plate as described above. Streptavidin and Pgp3 were dried onto the plates by incubation, uncovered, at 37 °C overnight. Sera/plasma was diluted 1:250. The following day the plates were rehydrated with washing buffer at room temperature for 15 min. Biotinylated peptides were diluted to 1 μg/ml in peptide coating buffer (0.1% bovine serum albumin (BSA) and 0.1% sodium azide in PBS at pH 7.5), and 50 μl/well added to the first 90 wells, blocking buffer was added to the remaining 6 wells of each plate. Peptides were bound to streptavidin at room temperature for 1 h with agitation. After 2 washes plates were blocked at room temperature for 30 min. After 2 washes the antibody binding and detection were performed as above.

Non-specific absorbance at OD 700 nm was subtracted from absorbance at 450 nm, samples in triplicate were averaged and values greater than 1 standard deviation from the mean excluded. If two out of three repeats were greater than 1 standard deviation from the mean, samples were retested. Values from each positive control dilution were averaged across all plates for each sample set. Values from each plate were divided by the averaged values, the mean deviation for each plate’s serial dilution from the average was used to transform each plate’s values.

### Statistical and *in silico* analyses

All comparisons were between scarring cases (TT and CO) and healthy matched controls unless otherwise stated. The intensity of responses was compared using a generalised linear model (glm) and number of positive responses using Fisher’s exact test. For the glm analysis, 10,000 permutations of the outcome variable were performed to generate an adjusted *P*-value (P*), controlling for type-1 error. Likelihood ratio tests were used to compare models with null models only including the covariates, age and gender.

Developmental cycle expression stage for each transcript was based on data and groupings from Belland et al. [45], this grouping was manually assigned to data from Nicholson et al. [46]. Localisation of expressed proteins was predicted using Cello [47], pSORTB [48] and loctree3 [49], three of the top performing prediction tools for bacterial proteins [49]. Predicted localisation was defined as the consensus from the 3 tools.

## Results

### Antibody responses are focussed on antigens expressed early and late during the developmental cycle and localised to interact with the host

**Fig. 1:**
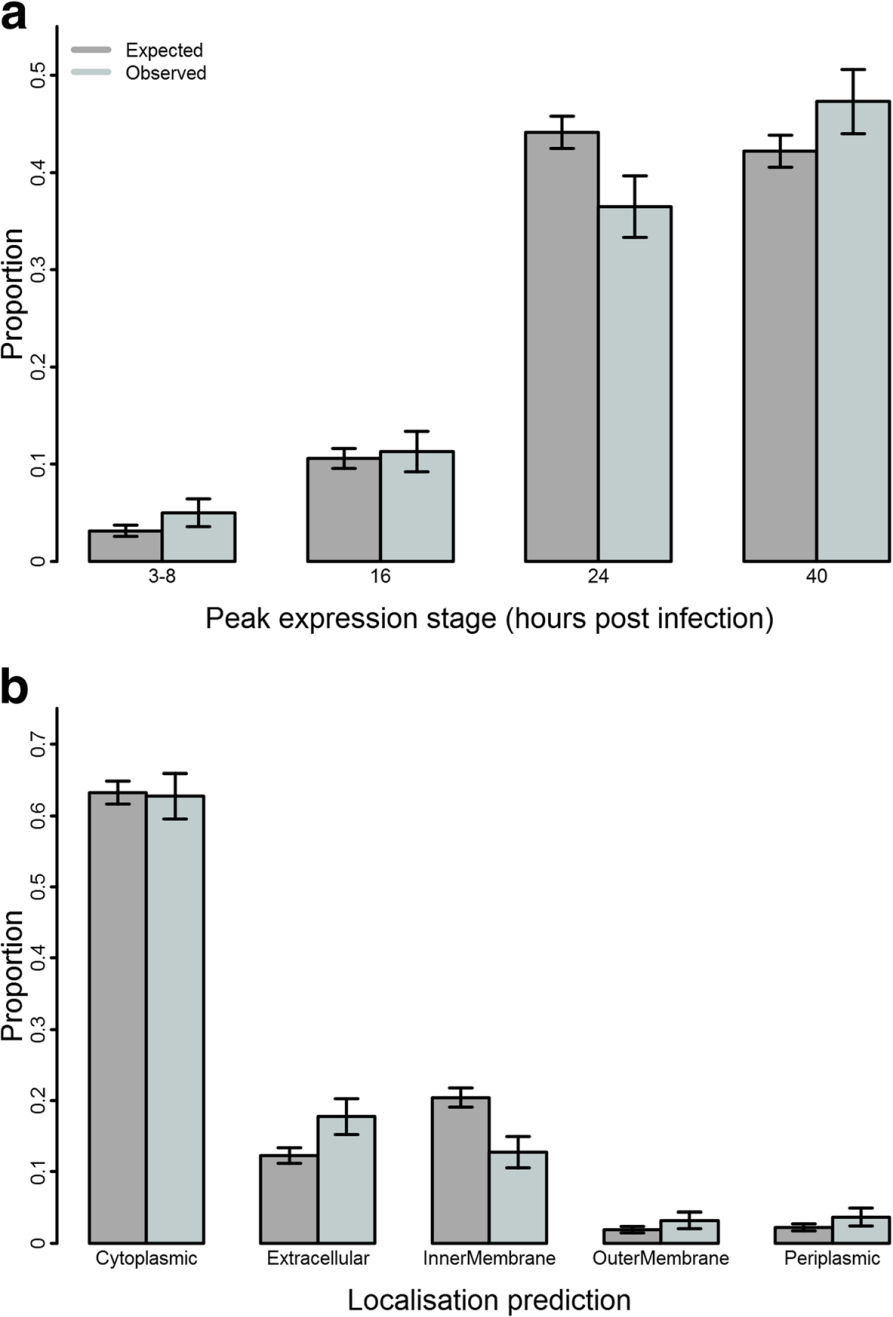
Over-representation of late and very early expressed proteins and extracellular, outer membrane and periplasmic proteins in immunogenic antigens. **a** Proteins identified through transcriptomics as expressed late or very early in the Ct developmental cycle were over-represented in the 230 immunogenic antigens (*light grey*) compared with the total unfiltered 908 (*dark grey*). **b** Proteins with a consensus localisation prediction of extracellular, outer membrane and periplasm were over-represented in the 230 immunogenic antigens (*light grey*) compared with the total unfiltered 908 (*dark grey*). Error bars represent standard deviation

After normalisation and filtering out of infrequently recognised antigens, responses of 59 individuals against 230 antigens were included in the analyses. Binary classification of positive and negative samples per antigen was achieved using the most appropriate clustering method, determined by average silhouette width (Additional file 3: Figure S1). Comparing the 230 antigens remaining after filtering with the 908 proteins screened on the array indicated, there was a significant over-representation of genes whose peak expression is either very early or very late in the developmental cycle, likely representing antigens important in cell entry and those exposed at exit, *P*-values 0.003 and 0.025 (Fig. 1a). Notably, proteins predicted to be extracellular/secreted, or localised to the outer membrane and periplasm were also over-represented in the immunogenic antigens, *P*-value < 0.001 (Fig. 1b).

**Fig. 2:**
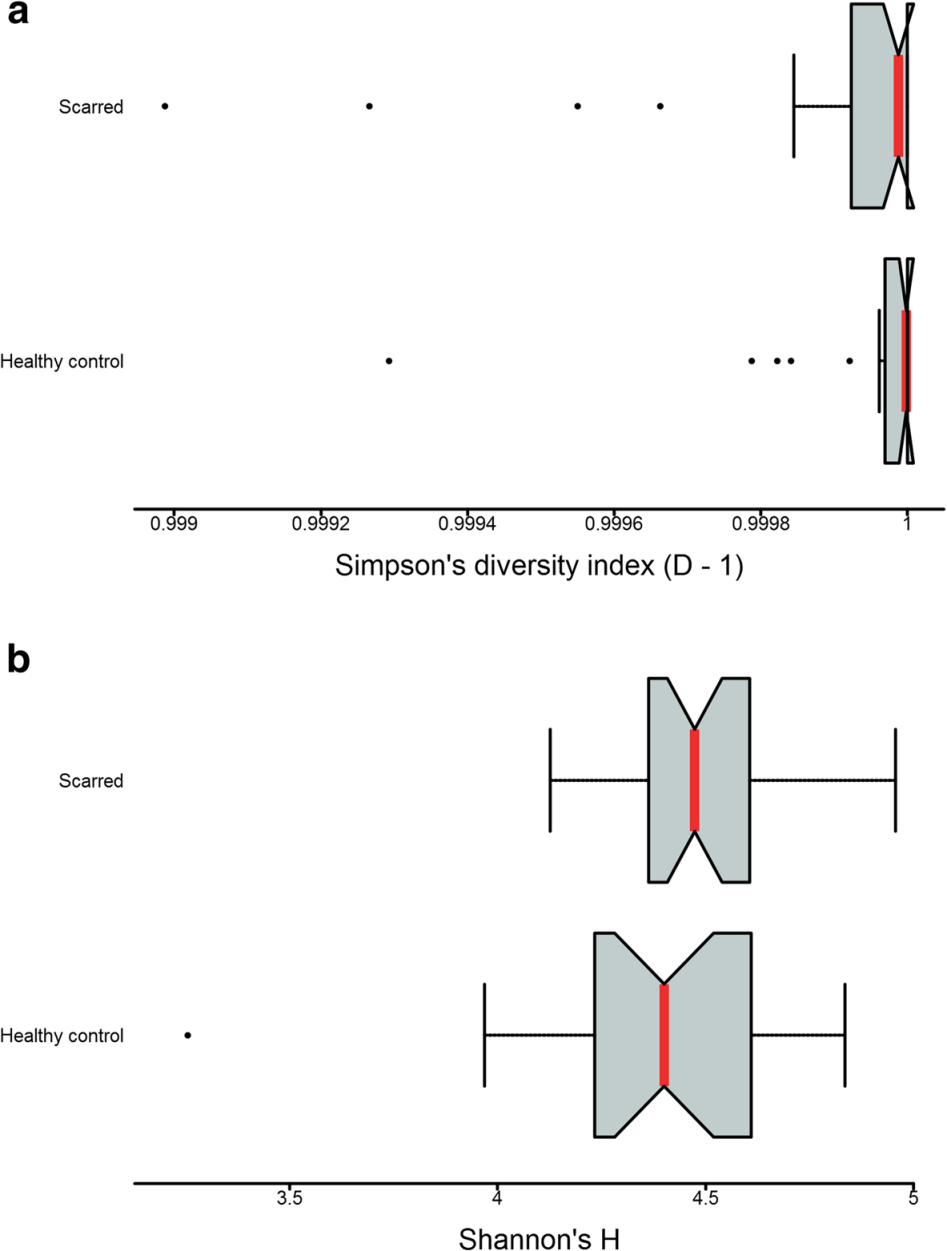
No significant differences in diversity of antibody responses between adults with and without scarring. Diversity was measured using Simpson’s diversity index (D-1) (**a**) and Shannon’s diversity index (H) (**b**). Median (*red lines*) and notches were calculated as the median +/- 1.57 x IQR/sqrt of n, where IQR is the interquartile range and n is the number of samples. The IQR times 1.5 was added to the 75th percentile and subtracted from the 25th percentile to determine the whiskers. Dots are outliers

Next, we utilised the global profile of antibody responses in scarred and healthy individuals to identify differences in diversity or evenness of responses. The breadth of response tended to be higher in scarred individuals however it was highly variable and did not reach significance, *P*-value 0.620. Simpson’s diversity index was skewed by a few individuals with very focused responses and showed no difference between groups, *P*-value = 0.451 (Fig. 2a). Shannon’s diversity index, similar to measures of breadth, tended to be lower in healthy individuals but the difference was not significant, *P*-value = 0.130 (Fig. 2b).

### Individual antibody responses are associated with conjunctival scarring

Association between participant’s evidence of conjunctival scarring and antibody responses against each antigen was determined using a generalised linear model adjusting for age and gender of the individuals. Nine differential antibody responses were identified between adults with and without scarring, permuted *P*-values ≤ 0.1 were permitted to include antigens outside the 95% distribution that were close to the P* = 0.05 threshold for significance (Table 4). All antigens except CT442 had higher responses in scarred individuals. Three of these antigens were identified by Lu et al. [31], nine antigens identified as differentially recognised by Lu et al. [31] were not supported by this re-analysis (Table 5).

**Table 4:** Differentially recognised antigens between adults with and without scarring. Univariate associations were determined using a generalised linear model. Variables were resampled 10,000 times and remodelled to determine permuted *P*-values (P*). The t-statistic (*t*) and its standard error, SE(*t*), are indicated. The odds ratio (OR) and 95% confidence interval (95% CI) of an individual having TT associated with a 1 unit increase in OD are indicated. Area under curve (AUC) for predicting TT status from a generalised linear model including each antigen are indicated

Antigen	*P*-value	P*	*t*	SE(*t*)	OR	95% CI	AUC
CT667	0.013	0.005	0.78	0.32	2.19	1.28–4.43	0.64
CT645	0.036	0.029	0.56	0.27	1.76	1.09–3.16	0.63
CT314	0.040	0.012	0.42	0.21	1.52	1.09–2.43	0.66
CT698	0.049	0.037	0.55	0.28	1.72	1.05–3.16	0.59
CT471	0.051	0.023	0.59	0.30	1.80	1.08–3.49	0.62
CT442	0.054	0.019	-0.12	0.06	0.89	0.77–0.98	0.63
CT679	0.057	0.011	0.66	0.35	1.94	1.11–4.28	0.61
CT425	0.070	0.049	0.51	0.28	1.66	1.04–3.16	0.54
CT706	0.098	0.064	0.32	0.19	1.37	1.03–2.19	0.57

**Table 5:** Agreement of differentially recognised antigens identified previously by Lu et al. [31] and in this study. The agreement, or disagreement, between the two analyses on antigens recognised more frequently or strongly by individuals with (scarred) or without (healthy) trachomatous scarring and trichiasis is indicated by ‘Yes’ or ‘No’ on the final column. Kappa = 0.25

Antigen	Lu et al. [31]	This study	Agreement
CT019	Healthy	None	No
CT117	Healthy	None	No
CT301	Healthy	None	No
CT314	None	Scarred	No
CT414	Scarred	None	No
CT425	None	Scarred	No
CT442	Healthy	Healthy	Yes
CT471	None	Scarred	No
CT553	Healthy	None	No
CT556	Healthy	None	No
CT571	Healthy	None	No
CT645	None	Scarred	No
CT667	Scarred	Scarred	Yes
CT679	None	Scarred	No
CT695	Healthy	None	No
CT698	None	Scarred	No
CT706	Scarred	Scarred	Yes
CT709	Healthy	None	No

### Independent follow-up of array-defined serological responses

To validate the antigens recognised in the microtitre plate array, three antigens were selected for further testing by ELISA. CT442 was selected for follow-up because of its potentially interesting intracellular biology as an inclusion membrane protein (Inc) known to induce T-cell responses and the only antigen with greater antibody responses in healthy individuals [50]. CT667 was selected as a homologue of CdsG, a conserved bacterial protein involved in type-three secretion (T3S) [51]. Homologues of CT667 in other bacteria act as a chaperone for the T3S-needle protein and in Ct CT667 has been localised to the host cytosol and around the inclusion membrane, depending on host cell type and stage of the Ct developmental cycle [52]. CT706 was selected as a homologue from *C. muridarum* and has previously been identified as immunogenic. These three were the only antigens identified as differentially recognised in the initial analysis [31], all of which were available as GST-fusion proteins. Pgp3 was included as an immunodominant positive control.

**Fig. 3:**
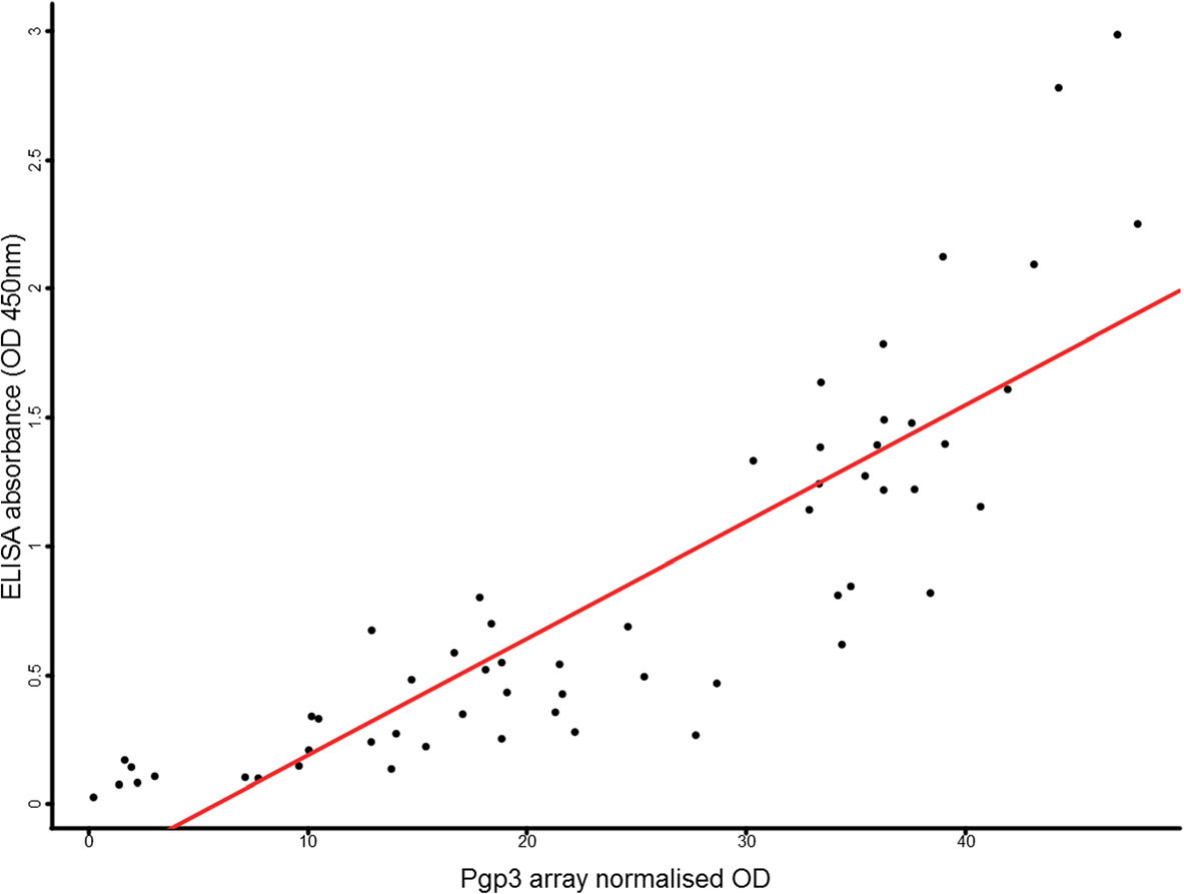
Pgp3 correlation between ELISA and array results. Responses to Pgp3 were retested in 59 arrayed serum using and in-house ELISA. Correlation between ELISA and array results was high. A linear model of results from the ELISA and array was used to fit the line (*red*)

The selected antigens were retested on 59 sera screened in the array, excluding one sample for which we no longer had serum, and the complete set of 116 sera available from the original study [31]. The positive control anti-Pgp3 results from the ELISA were strongly correlated with those from the array (rho = 0.91, *P*-value < 0.001) (Fig. 3).

**Fig. 4:**
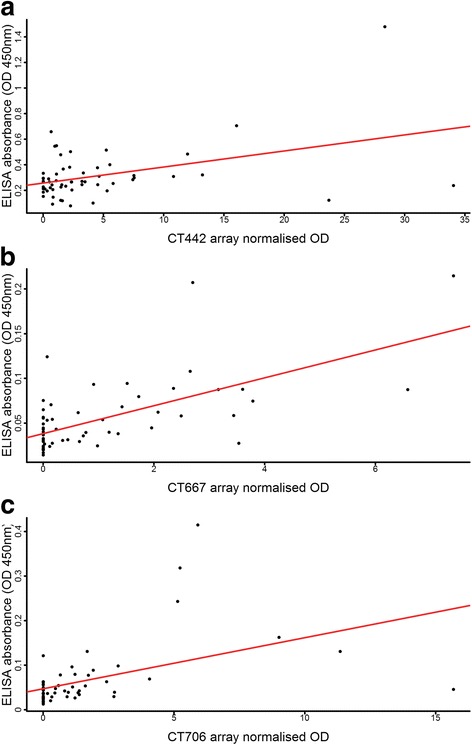
CT442, CT667 and CT706 correlation between ELISA and array results. Responses to CT442 (**a**), CT667 (**b**) and CT706 (**c**) were retested in 59 arrayed serum using an in-house ELISA. Correlation between ELISA and array results was poor for CT442 (rho 0.26, *P* = 0.046). Correlation between ELISA and array results for CT667 and CT706 were moderate (rho 0.59 and 0.58, both *P* < 0.001). A linear model of results from the ELISA and array was used to fit the line (*red*)

CT442, CT667 and CT706 had significant but weak correlations between array values and follow-up testing by ELISA (Fig. 4). All three trended towards the same healthy or scarring-association identified from the microarray. However, only CT667 was significantly associated with scarring (*P*-values; CT442 = 0.252, CT667 = 0.024 and CT706 = 0.169). None of these differentially recognised antigens was associated with healthy or scarred individuals when tested on the complete set of 116 samples, with p-values of 0.368, 0.169 and 0.289 respectively. The strongest responses to CT442 and CT667 were found in healthy individuals, but no clear association with protection was found.

### Independent serological testing of antibody targets associated with conjunctival scarring

These studies were cross-sectional collections of cases with scarring and matched controls. These sera were tested for antibody responses against the three differentially recognised antigens to determine if they were associated with scarring in independent studies from related trachoma-endemic communities.

All three antigens showed mixed or minimal association with scarring (Table 6). CT667 and CT706 antibody responses were not associated with scarring in either cohort. CT442 was significantly associated with scarring in adults from the Gambian study from 2011 which was inconsistent with its association with healthy adults from the microtitre plate array results.

**Table 6:** ELISA results from the complete 116 sera and two further scarring case-control studies. The three arrayed antigens were tested on the complete set of 116 sera (2006), 231 samples from a previous scarring case-control study in The Gambia (1995) and a subsequent scarring case-control study in The Gambia (2011). CT442 showed no association with the absence of scarring, and CT667/CT706 were not associated with scarring

Antigen	Sera	Healthy median (IQR)	Scarred median (IQR)	*P*-value	OR (95% CI)
CT442	2006	0.24 (0.19–0.33)	0.27 (0.18–0.33)	0.368	0.33 (0.02–3.36)
	1995	0.29 (0.18–0.43)	0.30 (0.15–0.46)	0.990	1.00 (0.46–2.13)
	2011	0.31 (0.22–0.38)	0.36 (0.24–0.48)	0.023	36.19 (2.20–1109.21)
CT667	2006	0.04 (0.03–0.06)	0.04 (0.03–0.07)	0.169	2766.28 (0.06–6.20 × 10^8^)
	1995	0.14 (0.05–0.23)	0.12 (0.03–0.23)	0.344	0.59 (0.18–1.68)
	2011	0.03 (0.02–0.06)	0.03 (0.02–0.07)	0.802	0.23 (2.47 × 10^-6^–24,634.90)
CT706	2006	0.05 (0.03–0.07)	0.04 (0.03–0.06)	0.289	42.59 (0.07–1.26 × 10^5^)
	1995	0.14 (0.06–0.23)	0.12 (0.04–0.23)	0.307	0.58 (0.18–1.57)
	2011	0.02 (0.02–0.06)	0.03 (0.02–0.08)	0.940	1.24 (0.005–619.41)

## Discussion

In this study results from a microplate array used to screen sera from a case-control study of Gambian adults with scarring trachoma for Ct-specific antibody responses were re-analysed. We found that antibody responses were focussed on secreted, outer membrane and periplasmic proteins. There was a trend towards less diverse global antibody responses in adults without conjunctival scarring. Heightened responses to 8 antigens were associated with the presence of scarring, only antibody responses to CT442 were associated with a lack of scarring. Three of these differentially recognised antigens were concordant with previous analysis of this micro-plate array. Association of antibody responses against these three antigens with scarring was assessed by further independent serological testing. CT667 and CT706 were not associated with scarring in adults in independent case-control studies from The Gambia. CT442 responses were not associated with an absence of scarring in adults in the independent studies.

### Global antibody profiles indicate common targeting of responses in adults

The number of immunogenic antigens, those that passed initial filtering, and the number of commonly recognised antigens, determined by breadth, were both low. This likely reflects the reduced frequency and duration of Ct infection and active trachomatous disease in older individuals. It also suggests a time dependent focusing of antibody responses. These individuals experience a lifetime of exposure to Ct promoting selective activation and maintenance of high-affinity antibodies. As frequency and duration of Ct infection decrease, the opportunity for restimulation of Ct-specific plasma cells may be reduced. While some of these Ct-specific cells may be maintained as long-lived plasma cells, reactivation by abundant and immunogenic Ct antigens favours survival and expansion of specific plasma cells. This can put antigens commonly exposed to the extracellular space and B-cells at a selective advantage. Antibodies specific to these antigens are more likely to be activated during infrequent episodes of Ct infection, promoting their activation and affinity maturation. This more focused profile of antibody responses was reflected in the common recognition of antigens expressed at similar stages of chlamydial development or their cellular localisation.

The recognition of proteins expressed very early and late in the Ct developmental cycle was over-represented. It is during these periods that Ct, in the form of infectious EBs, is most exposed to the humoral immune system. Potential targets may be EB outer membrane proteins, proteins in the membrane of extrusions [53] and a more unpredictable group of proteins exposed to the extracellular environment upon Ct lytic exit from cells. This was supported by the over-representation of the chlamydial outer membrane, extracellular and periplasmic proteins in the complete list of immunogenic antigens.

The diversity of antibody responses was not significantly different between adults with and without trachomatous scarring. There was a trend towards increased diversity in scarred adults by Shannon’s diversity index, but this was not supported by Simpson’s diversity index. This reinforces the finding that adults, in general, had focussed antibody responses.

### Few antigens are differentially recognised between individuals with and without scarring trachoma

There was no difference in the diversity profile of antibody responses between adults with and without scarring. However, a small panel of antigens was differentially recognised between these groups. Antibody responses to eight antigens were associated with scarring and responses to a single antigen were associated with a lack of scarring. The low number of differentially recognised antigens supports a focusing and reduced heterogeneity of antibody responses in older individuals. Three of these antigens were identified as differentially recognised in the analysis by Lu et al. [31]: CT442, CT667 and CT706. Of the seven antigens previously identified that were not supported following independent analysis, six had been originally differentially associated by Fisher’s exact test comparing the number of positive responses between adults with and without scarring. A more objective definition of positivity was used in the follow-up independent analysis leading to no significant differentially recognised antigens being identified.

Limited published information was available for these antigens. CT314, CT425 and CT442 have been identified as immunogenic in patients with urogenital Ct or related disease [25], and mouse models have shown CT442 is a target of CD8^+^ T-cell responses [50]. No functional studies of these antigens have been undertaken, although some of them share homology with bacterial proteins of known functions. These relate to cell division (CT471 and CT697), transcription (CT314), protein quality control (CT706) and type-3 secretion (CT667). These homologues and localisation predictions suggest they likely reside within the inclusion, meaning they would only be exposed to B-cells upon cell lysis. There is evidence that some of these targets may be more easily accessible to the host immune system. Proteomic analysis of Ct EBs identified CT314 as a component of the outer membrane complex [54]. CT442 has been localised to the inclusion membrane [50] at early stages of the Ct developmental cycle, CT667 appears to reside in the cytosol [52].

### Validation of large-scale array-based methods is essential

Responses against the positive control Pgp3 were strongly correlated between the array and in-house ELISA. The correlation was strong for the differentially recognised CT667 and CT706 but not CT442, although the latter was tested using a short peptide rather than a full-length recombinant. A caveat to the lack of identification of some immunodominant antigens on the array, such as MOMP and the majority of polymorphic membrane proteins (pmp) is their poor immunogenicity since they did not pass initial filtering of recognised antigens. PmpC (CT414) and PmpD (CT812) were the only Pmp’s frequently recognised and both were expressed as fragments, not full-length proteins. This suggests large, multimeric proteins such as these should be expressed as fragments or peptides alongside full-length proteins in microarrays.

Antibody responses associated with conjunctival scarring (CT667 and CT706) or an absence of conjunctival scarring (CT442) from the array analysis were not supported in the complete set of sera or the two further sets of sera. These results suggest the differentially recognised antigens identified are likely false-positive artefacts from screening of small numbers of sera on the array. CT442 is an example of where one or two individuals with high levels of antibody against an antigen skewed the results in the small data set. This highlights the need for validation and follow-up of antibody targets identified through related high-throughput techniques.

## Conclusions

Antibody responses against Ct antigens were focussed in adults. There was considerable homogeneity between those with and without conjunctival scarring. Antibody responses were focussed on antigens expressed early and late in the Ct developmental cycle and localised to the outer membrane or secreted. Only nine antigens were differentially recognised between the two groups, of which only one (CT442) was inconsistently associated with a lack of scarring. Understanding how these antigens become antibody targets and how this could impact scarring is difficult due to limited localisation and functional information. Heterogenous responses in the studies examined here highlight the need for large sample sizes and clearly defined clinical phenotypes in human studies of humoral immunity to reduce the influence of outliers. Immunogenetic information would further aid understanding of the observed heterogeneity in antibody responses.

## Additional files

Additional file 1: Table S1. Summary of previous Ct micro-array antigen identification. (DOCX 16 kb)
Additional file 2: Table S2. Raw OD values and anonymised clinical data from the microarray. (XLSX 290 kb)
Additional file 3: Figure S1. Average silhouette widths for clustering method trialled for 230 antigens. ‘Best’ method had the highest median across all antigens. Clustering methods are detailed on the left-hand side. Red lines indicate the median. Notches were calculated as median +/- 1.57 × IQR/sqrt of n, where IQR is the interquartile range and n is the number of samples. The whiskers were calculated by adding 1.5 times the IQR to the 75 percentile and subtracting 1.5 times the IQR from the 25 percentile. Dots are outliers. (TIFF 337 kb)

## References

[1] Mabey DCW, Solomon AW, Foster A (2003). Trachoma Lancet.

[2] Grayston JT, Wang S-P, Yeh LJ, Kuo CC (1985). Importance of reinfection in the pathogenesis of trachoma. Rev Infect Dis.

[3] Silverstein AM (1973). The immunologic modulation of infectious disease pathogenesis. Invest Ophthalmol.

[4] Burton MJ, Rajak SN, Bauer J, Weiss HA, Tolbert SB, Shoo A (2011). Conjunctival transcriptome in scarring trachoma. Infect Imm.

[5] Taylor HR, Siler JA, Mkocha HA, Munoz B, West S (1992). The natural history of endemic trachoma: a longitudinal study. Am J Trop Med Hyg.

[6] Burton MJ, Bowman RJ, Faal H, Aryee EA, Ikumapayi UN, Alexander ND (2006). The long-term natural history of trachomatous trichiasis in the Gambia. Invest Ophthalmol Vis Sci.

[7] Ramadhani AM, Derrick T, Holland MJ, Burton MJ (2016). Blinding trachoma: Systematic review of rates and risk fctors for progressive disease. PLoS NTDs.

[8] Mabey DC, Bailey RL, Ward ME, Whittle HC (1992). A longitudinal study of trachoma in a Gambian village: implications concerning the pathogenesis of chlamydial infection. Epidemiol Infect.

[9] Burton MJ, Holland MJ, Jeffries D, Mabey DCW, Bailey RL. Conjunctival chlamydial 16S ribosomal RNA expression in trachoma: Is chlamydial metabolic activity required for disease to develop? Clin Infect Dis. 2006;42(4):463–370. 10.1086/499814 16421789

[10] Burton MJ, Adegbola RA, Kinteh F, Ikumapayi UN, Foster A, Mabey DC (2007). Bacterial infection and trachoma in the Gambia: a case control study. Invest Ophthalmol Vis Sci.

[11] Hu V, Massae P, Weiss HA, Chevallier C, Onyango JJ, Afwamba IA (2011). Bacterial infection in scarring trachoma. Invest Ophthalmol Vis Sci.

[12] Guzey M, Ozardali I, Kilic A, Basar E, Dogan Z, Satici A (2001). The treatment of severe trachomatous dry eye with canalicular silicone plugs. Eye.

[13] Lucena A, Akaishi PM, Rodrigues Mde L, Cruz AA (2012). Upper eyelid entropion and dry eye in cicatricial trachoma without trichiasis. Arq Bras Oftalmol.

[14] Ward M, Bailey R, Lesley A, Kajbaf M, Robertson J, Mabey D (1990). Persisting inapparent chlamydial infection in a trachoma endemic community in The Gambia. Scand J Infect Dis Supplementum.

[15] Peeling RW, Bailey RL, Conway DJ, Holland MJ, Campbell AE, Jallow O (1998). Antibody response to the 60-kDa chlamydial heat-shock protein is associated with scarring trachoma. J Infect Dis.

[16] Holland MJ, Bailey RL, Hayes LJ, Whittle HC, Mabey DCW (1993). Conjunctival scarring in trachoma is associated with depressed cell-mediated immune responses to chlamydial antigens. J Infect Dis.

[17] Hessel T, Dhital SP, Plank R, Dean D (2001). Immune response to chlamydial 60-kilodalton heat shock protein in tears from Nepali trachoma patients. Infect Imm.

[18] Skwor T, Kandel RP, Basravi S, Khan A, Sharma B, Dean D (2010). Characterization of humoral immune responses to chlamydial HSP60, CPAF, and CT795 in inflammatory and severe trachoma. Invest Ophthalmol Vis Sci.

[19] Davies DH, Liang X, Hernandez JE, Randall A, Hirst S, Mu Y (2005). Profiling the humoral immune response to infection by using proteome microarrays: high-throughput vaccine and diagnostic antigen discovery. Proc Natl Acad Sci USA.

[20] Doolan DL, Mu Y, Unal B, Sundaresh S, Hirst S, Valdez C (2008). Profiling humoral immune responses to *P. falciparum* infection with protein microarrays. J. Proteomics.

[21] Trieu A, Kayala MA, Burk C, Molina DM, Freilich DA, Richie TL (2011). Sterile protective immunity to malaria is associated with a panel of novel *P. falciparum* antigens. Mol Cell Proteomics.

[22] Kunnath-Velayudhan S, Salamon H, Wang HY, Davidow AL, Molina DM, Huynh VT (2010). Dynamic antibody responses to the *Mycobacterium tuberculosis* proteome. Proc Natl Acad Sci USA.

[23] Liang L, Felgner PL (2015). A systems biology approach for diagnostic and vaccine antigen discovery in tropical infectious diseases. Curr Opin Infect Dis.

[24] Sharma J, Zhong Y, Dong F, Piper JM, Wang G, Zhong G (2006). Profiling of human antibody responses to *Chlamydia trachomatis* urogenital tract infection using microplates arrayed with 156 chlamydial fusion proteins. Infect Imm.

[25] Wang J, Zhang Y, Lu C, Lei L, Yu P, Zhong G (2010). A genome-wide profiling of the humoral immune response to *Chlamydia trachomatis* infection reveals vaccine candidate antigens expressed in humans. J Immunol.

[26] Rodgers AK, Budrys NM, Gong S, Wang J, Holden A, Schenken RS (2011). Genome-wide identification of *Chlamydia trachomatis* antigens associated with tubal factor infertility. Fertil Steril.

[27] Budrys NM, Gong S, Rodgers AK, Wang J, Louden C, Shain R (2012). *Chlamydia trachomatis* antigens recognized in women with tubal factor infertility, normal fertility, and acute infection. Obstet Gynecol.

[28] Coler RN, Bhatia A, Maisonneuve JF, Probst P, Barth B, Ovendale P (2009). Identification and characterization of novel recombinant vaccine antigens for immunization against genital *Chlamydia trachomatis*. FEMS Immunol Med Microbiol.

[29] Finco O, Frigimelica E, Buricchi F, Petracca R, Galli G, Faenzi E (2011). Approach to discover T- and B-cell antigens of intracellular pathogens applied to the design of *Chlamydia trachomatis* vaccines. Proc Natl Acad Sci USA.

[30] Teng A, Cruz-Fisher MI, Cheng C, Pal S, Sun G, Ralli-Jain P (2012). Proteomic identification of immunodominant chlamydial antigens in a mouse model. J Proteomics.

[31] Lu C, Holland MJ, Gong S, Peng B, Bailey RL, Mabey DW (2012). Genome-wide identification of *Chlamydia trachomatis* antigens associated with trachomatous trichiasis. Invest Ophthalmol Vis Sci.

[32] Stekel D (2003). Microarray Bioinformatics.

[33] Conway DJ, Holland MJ, Campbell AE, Bailey RL, Krausa P, Peeling RW (1996). HLA Class I and II polymorphisms and trachomatous scarring in a *Chlamydia trachomatis*-endemic population. J Infect Dis.

[34] Conway DJ, Holland MJ, Bailey RL, Campbell AE, Mahdi OSM, Jennings R, et al. Scarring trachoma is associated with polymorphism in the tumor necrosis factor alpha (TNF-alpha) gene promoter and with elevated TNF-alpha levels in tear fluid. Infect Imm. 1997;65(3):1003–6. 10.1128/iai.65.3.1003-1006.1997 PMC175081 9038309

[35] Derrick T, Roberts CH, Rajasekhar M, Burr SE, Joof H, Makalo P (2013). Conjunctival microRNA expression in inflammatory trachomatous scarring. PLoS NTDs.

[36] Kroll TC, Wolfl S (2002). Ranking: a closer look on globalisation methods for normalisation of gene expression arrays. Nucleic Acids Res.

[37] Migchelsen SJ, Martin DL, Southisombath K, Turyaguma P, Heggen A, Rubangakene PP (2016). Defining seropositivity thresholds for use in trachoma elimination studies. PLoS NTDs.

[38] Rousseeuw PJ (1987). Silhouettes: A graphical aid to the interpretation and validation of cluster analysis. J Comput Appl Math.

[39] Magurran AE. Assumptions of biodiversity measurement. In: Measuring Biological Diversity. MA, USA: Blackwell Science; 2004. p. 256.

[40] Gotelli NJ, Chao A, Levin S (2013). Measuring and estimating species richness, species diversity, and biotic similarity from sampling data. Encyclopedia of Biodiversity.

[41] Morris EK, Caruso T, Buscot F, Fischer M, Hancock C, Maier TS (2014). Choosing and using diversity indices: insights for ecological applications from the German Biodiversity Exploratories. Evol Ecol.

[42] Rahman KS, Chowdhury EU, Poudel A, Ruettger A, Sachse K, Kaltenboeck B (2015). Defining species-specific immunodominant B cell epitopes for molecular serology of *Chlamydia* species. Clin Vaccine Immunol.

[43] Wills GS, Horner PJ, Reynolds R, Johnson AM, Muir DA, Brown DW (2009). Pgp3 antibody enzyme-linked immunosorbent assay, a sensitive and specific assay for seroepidemiological analysis of *Chlamydia trachomatis* infection. Clin Vaccine Immunol.

[44] Goodhew EB, Priest JW, Moss DM, Zhong G, Munoz B, Mkocha H (2012). CT694 and pgp3 as serological tools for monitoring trachoma programs. PLoS NTDs.

[45] Belland RJ, Zhong G, Crane DD, Hogan D, Sturdevant D, Sharma J (2003). Genomic transcriptional profiling of the developmental cycle of *Chlamydia trachomatis*. Proc Natl Acad Sci USA.

[46] Nicholson TL, Olinger L, Chong K, Schoolnik G, Stephens RS (2003). Global stage-specific gene regulation during the developmental cycle of *Chlamydia trachomatis*. J Bacteriol.

[47] Yu CS, Lin CJ, Hwang JK (2004). Predicting subcellular localization of proteins for Gram-negative bacteria by support vector machines based on n-peptide compositions. Protein Sci.

[48] Yu NY, Wagner JR, Laird MR, Melli G, Rey S, Lo R (2010). PSORTb 3.0: improved protein subcellular localization prediction with refined localization subcategories and predictive capabilities for all prokaryotes. Bioinformatics.

[49] Goldberg T, Hecht M, Hamp T, Karl T, Yachdav G, Ahmed N (2014). LocTree3 prediction of localization. Nucleic Acids Res.

[50] Starnbach MN, Loomis WP, Ovendale P, Regan D, Hess B, Alderson M (2003). An inclusion membrane protein from *Chlamydia trachomatis* enters the MHC Class I pathway and stimulates a CD8^+^ T cell response. J Immunol.

[51] Betts HJ, Twiggs LE, Sal MS, Wyrick PB, Fields KA (2008). Bioinformatic and biochemical evidence for the identification of the type III secretion system needle protein of *Chlamydia trachomatis*. J Bacteriol.

[52] Spedding L (2009). Novel effector protein secretion and transcriptional regulation of the Type Three Secretion System in *Chlamydia trachomatis* (Doctoral dissertation). Molecular Biosciences.

[53] Liu X, Afrane M, Clemmer DE, Zhong G, Nelson DE (2010). Identification of *Chlamydia trachomatis* outer membrane complex proteins by differential proteomics. J Bacteriol.

[54] Bartolini, E., E. Ianni, E. Frigimelica, R. Petracca, G. Galli, F. Berlanda Scorza, et al. Recombinant outer membrane vesicles carrying *Chlamydia muridarum* HtrA induce antibodies that neutralize chlamydial infection in vitro. J Extracell Vesicles. 2013;2 10.3402/jev.v2i0.20181 PMC3760637 24009891

