# Supplementary material for: Profiling and validation of individual and patterns of Chlamydia trachomatis-specific antibody responses in trachomatous trichiasis
Source: Parasit Vectors. 2017 Mar 13;10:143. doi: 10.1186/s13071-017-2078-8 (PMC5347170; doi:10.1186/s13071-017-2078-8)
Supplement: Additional file 1: Table S1. — Summary of previous Ct micro-array antigen identification. (DOCX 16 kb) [file 13071_2017_2078_MOESM1_ESM.docx]

Supplementary Table 1. Summary of previous Ct micro-array antigen identification.

| ID | Name | T-cell recognition | B-cell recognition |
| --- | --- | --- | --- |
| CT004 | GatB | 1 | 0 |
| CT015 | PhoH | 1 | 0 |
| CT016 | Hypothetical | 1 | 0 |
| CT019 | IleS | 0 | 1 |
| CT022 | RpmE2 | 0 | 1 |
| CT035 | BPL | 1 | 0 |
| CT043 | Hypothetical | 2 | 0 |
| CT049 | Hypothetical | 0 | 1 |
| CT067 | YtgA | 0 | 2 |
| CT082 | Hypothetical | 0 | 1 |
| CT089 | CopN | 0 | 6 |
| CT101 | Hypothetical | 0 | 1 |
| CT110 | GroEL1 (HSP60) | 1 | 4 |
| CT111 | GroES | 1 | 0 |
| CT114 | Hypothetical | 1 | 0 |
| CT116 | IncE | 0 | 1 |
| CT117 | IncF | 0 | 1 |
| CT118 | IncG | 0 | 1 |
| CT119 | IncA | 1 | 4 |
| CT142 | Hypothetical | 0 | 2 |
| CT143 | Hypothetical | 0 | 2 |
| CT147 | Hypothetical | 0 | 4 |
| CT153 | Hypothetical | 1 | 2 |
| CT168 | Hypothetical | 1 | 0 |
| CT184 | YqgF | 1 | 0 |
| CT226 | Hypothetical | 0 | 1 |
| CT228 | Hypothetical | 0 | 1 |
| CT240 | RecR | 0 | 1 |
| CT255 | Hypothetical | 1 | 0 |
| CT279 | Nqr3 | 1 | 0 |
| CT301 | PknD | 0 | 1 |
| CT315 | RpoB | 1 | 0 |
| CT316 | R17 | 0 | 1 |
| CT322 | TuEF | 1 | 3 |
| CT341 | DnaJ | 1 | 0 |
| CT342 | Rs21 | 1 | 0 |
| CT355 | Hypothetical | 0 | 1 |
| CT372 | Hypothetical | 1 | 0 |
| CT376 | MdhC | 0 | 2 |
| CT381 | ArtJ | 0 | 4 |
| CT396 | DnaK (HSP70) | 2 | 1 |
| CT414 | PmpC | 0 | 2 |
| CT415 | YebL | 0 | 1 |
| CT442 | CrpA | 0 | 4 |
| CT443 | OmcB | 3 | 6 |
| CT456 | TARP | 0 | 3 |
| CT460 | SWIB | 1 | 0 |
| CT480 | DppA | 1 | 0 |
| CT492 | YacE | 1 | 0 |
| CT509 | RS13 | 1 | 0 |
| CT529 | Cap1 | 0 | 3 |
| CT553 | Fmu | 0 | 1 |
| CT556 | Hypothetical | 0 | 1 |
| CT557 | LpdA | 0 | 2 |
| CT559 | YscJ | 0 | 1 |
| CT571 | GspE | 0 | 1 |
| CT587 | Eno | 1 | 0 |
| CT589 | Hypothetical | 0 | 1 |
| CT600 | Pal | 1 | 0 |
| CT601 | PapQ | 1 | 0 |
| CT603 | TSA | 1 | 1 |
| CT611 | Hypothetical | 1 | 0 |
| CT619 | Hypothetical | 0 | 1 |
| CT622 | CHLPN homologue | 0 | 1 |
| CT667 | Hypothetical | 0 | 1 |
| CT681 | MOMP | 2 | 5 |
| CT694 | Hypothetical | 1 | 3 |
| CT695 | Hypothetical | 0 | 3 |
| CT702 | Hypothetical | 0 | 1 |
| CT706 | ClpP2 | 0 | 1 |
| CT709 | MreB | 0 | 1 |
| CT711 | Hypothetical | 1 | 0 |
| CT716 | Hypothetical | 1 | 0 |
| CT733 | Hypothetical | 1 | 0 |
| CT734 | Hypothetical | 1 | 0 |
| CT755 | GroEL3 | 1 | 1 |
| CT795 | Hypothetical | 0 | 3 |
| CT798 | GlgA | 0 | 2 |
| CT806 | Ptr | 0 | 2 |
| CT812 | PmpD | 1 | 2 |
| CT813 | Hypothetical | 0 | 2 |
| CT823 | HtrA | 1 | 2 |
| CT828 | NrdB | 0 | 1 |
| CT841 | FtsH | 0 | 1 |
| CT858 | CPAF | 1 | 4 |
| CT866 | GlgB | 0 | 1 |
| CT871 | PmpG | 1 | 0 |
| CT872 | PmpH | 1 | 0 |
| CT875 | Hypothetical | 1 | 3 |
| pCT03 | Pgp3 | 0 | 2 |

Ct D/UW3 nomenclature was used. T and B-cell recognition numbers were the how often they were found to be immunogenic in 6 previous Ct micro-arrays.
