# Supplementary figures and images for: Profiling and validation of individual and patterns of Chlamydia trachomatis-specific antibody responses in trachomatous trichiasis
Source: Parasit Vectors. 2017 Mar 13;10:143. doi: 10.1186/s13071-017-2078-8 (PMC5347170; doi:10.1186/s13071-017-2078-8)

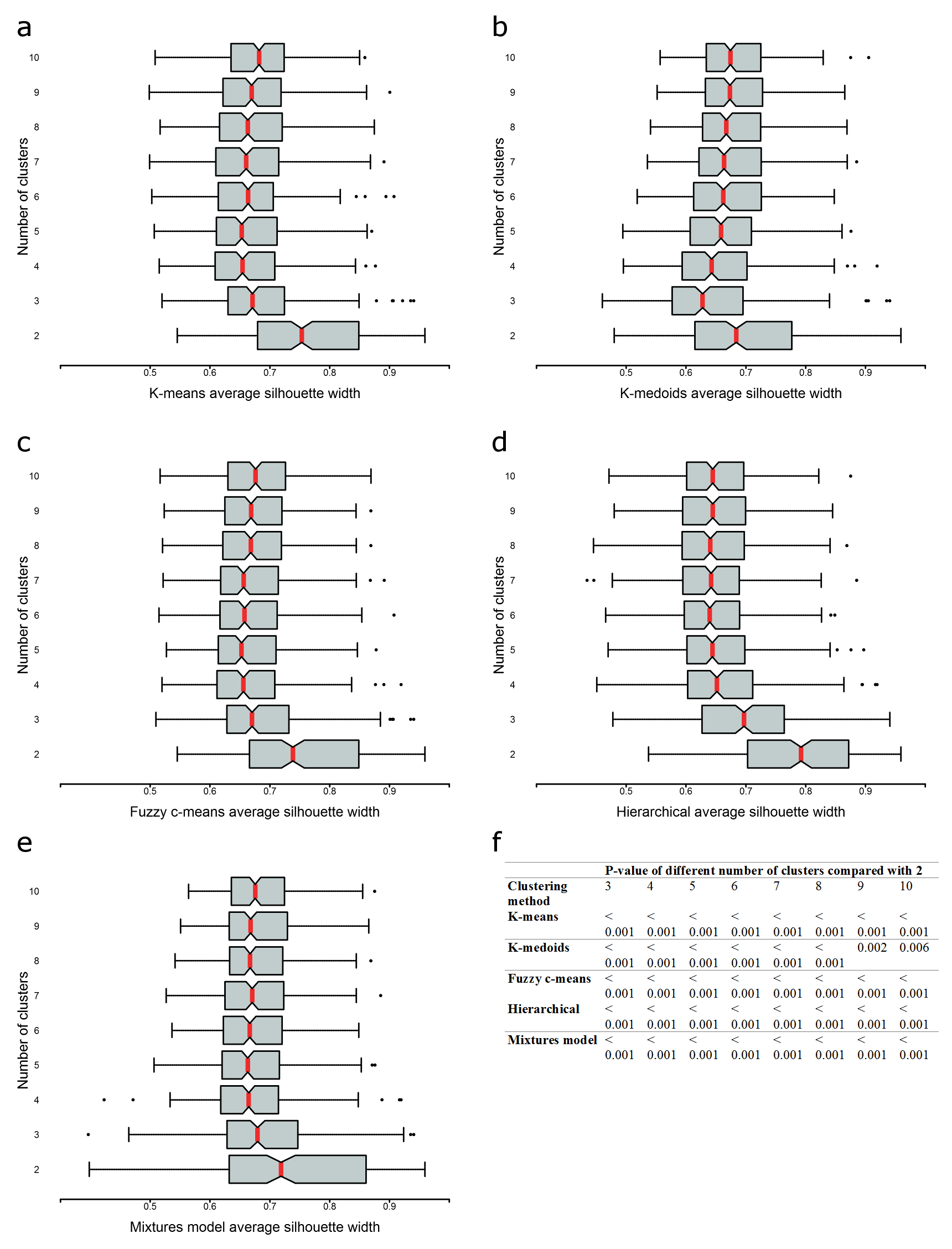

Supplement: Additional file 3: Figure S1. — Average silhouette widths for clustering method trialled for 230 antigens. ‘Best’ method had the highest median across all antigens. Clustering methods are detailed on the left-hand side. Red lines indicate the median. Notches were calculated as median +/- 1.57 × IQR/sqrt of n, where IQR is the interquartile range and n is the number of samples. The whiskers were calculated by adding 1.5 times the IQR to the 75 percentile and subtracting 1.5 times the IQR from the 25 percentile. Dots are outliers. (TIFF 337 kb) [file 13071_2017_2078_MOESM3_ESM.tiff]
